# Fear of the Relapse: Effect of Composite Type on Adhesion Efficacy of Upper and Lower Orthodontic Fixed Retainers: In Vitro Investigation and Randomized Clinical Trial

**DOI:** 10.3390/polym12040963

**Published:** 2020-04-21

**Authors:** Andrea Scribante, Simone Gallo, Benedetta Turcato, Federico Trovati, Paola Gandini, Maria Francesca Sfondrini

**Affiliations:** Unit of Orthodontics and Paediatric Dentistry, Section of Dentistry, Department of Clinical, Surgical, Diagnostic and Paediatric Sciences, University of Pavia, 27100 Pavia, Italy; benedetta.turcato01@universitadipavia.it (B.T.); federico.trovati01@universitadipavia.it (F.T.); paola.gandini@unipv.it (P.G.); francesca.sfondrini@unipv.it (M.F.S.)

**Keywords:** fixed retention, multistrand wire, retainer, splint, adhesion, bonding, resin, orthodontic composite, flow, nanocomposite, shear, clinical trial

## Abstract

The aim of this laboratory and clinical study is to determine the reliability of the flowable nanocomposite Filtek Supreme XTE (FL) for the adhesion of orthodontic retainers, compared to highly filled orthodontic resin Transbond XT (XT). Portions of a round section multistranded wire (Ortosmail Krugg) were bonded to 40 bovine incisors with Scotchbond Universal in total-etch modality. For group one (XT, 20 samples), the orthodontic resin was used, whereas in group two (FL, 20 samples), the flowable one. Specimens were placed into a universal testing machine which applied a shear force on retainers with a crosshead speed of one/minute. Shear bond strength (SBS) and adhesive remnant index (ARI) scores were calculated. In the clinical trial, 100 patients requiring a canine-to-canine palatal and lingual retainer were randomly divided into two groups, according to the resin used for bonding procedure: the orthodontic in group one (XT, 50 participants) and the flowable in group two (FL, 50 participants). Monthly visits were carried out over a 24-month follow up to assess any detachment occurring on teeth of both arches. All data were submitted to statistical analysis. In vitro, FL reported a significant lower mean SBS, whereas no significant differences in ARI were reported between the two groups which both showed a major frequency of scores “1” and “2”. At the end of the 24-month follow up, FL reported significantly higher failure rates in both arches besides a significantly lower survival rate starting from the sixth month after retainers bonding. According to the results assessed in vitro and clinically, XT would be preferable to FL when performing retainers bonding procedure.

## 1. Introduction

**Figure 1:**
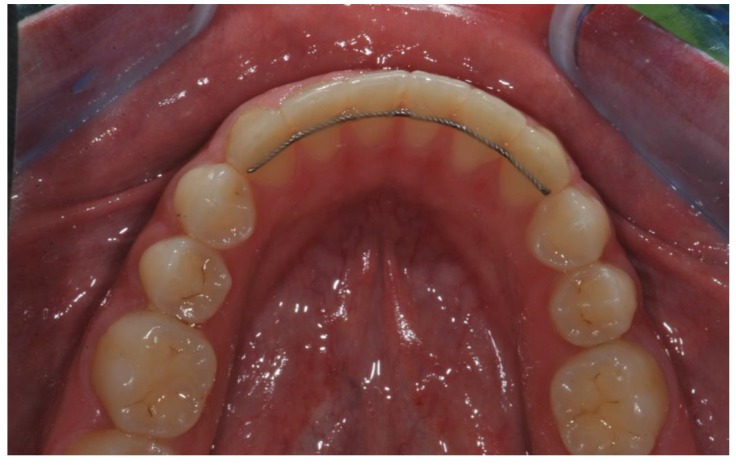
Orthodontic fixed retainer immediately after positioning.

The goal of orthodontic treatment is to move dental elements and correct malocclusions. However, it has been shown that at the end of the therapy, teeth tend to regain their pre-treatment position independently [1]. For this reason, retention is considered the final and essential step to maintain, through an adequate appliance, the correct position reached by dental elements at the end of the active orthodontic therapy. Clinicians agree that retention should be maintained as long as a perfect alignment is desired [2]. Several studies investigated the most used retention device in various European and non-European countries, and bonded retainers resulted to be the most widely used device in different countries, especially for the mandibular arch [3,4,5,6,7,8]. This is due to their reliability, independence of patient compliance, high effectiveness, simplicity of application, almost invisibility (Figure 1) and well acceptance by patients [9].

**Figure 2:**
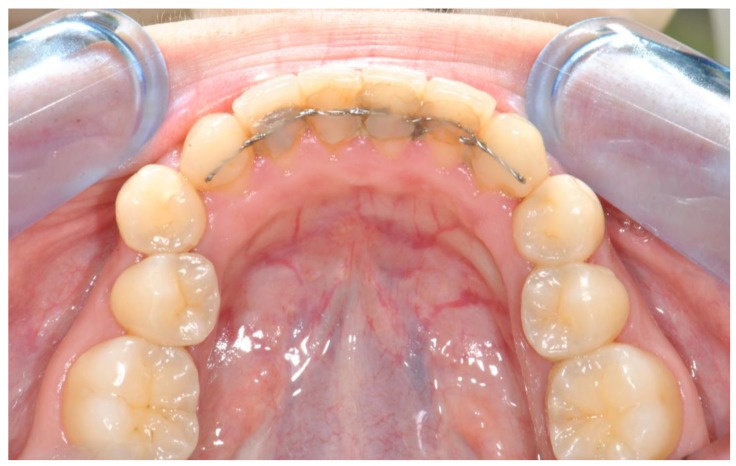
Detached and infiltrated orthodontic fixed retainer.

The adoption of reliable bonding techniques is fundamental in the field of bonded retainers in order to avoid the risk of detachment (Figure 2), which is the major concern linked to these appliances for both patients and orthodontists. 

In restorative dentistry, it is believed that considerable benefits are obtained from etching enamel with phosphoric acid prior to the application of a universal adhesive system: the results of various studies show, indeed, that the bonding force (shear bond strength) of these adhesives increases considerably following pre-etching of the enamel [10,11,12,13,14,15,16,17,18,19,20]. We have found no previous research testing this methodology in orthodontics.

Along with the adhesive, different resin composites can be chosen to be applied around the retainer to perform bonding. According to in vitro studies on bonding strength, the detachment of a splinted wire is more frequently of cohesive type, taking place at the interface between the wire and the composite [18,21,22,23]. Therefore, proper resins should be used in order to increase the bond strength with the wires, avoiding the risk of failure with a possible orthodontic relapse. 

The purpose of the present in vitro and clinical study is to analyze the efficacy of a flowable composite, generally employed in restorative dentistry, if used in orthodontics to bond fixed retainers through a universal adhesive applied after enamel pre-etching (total-etch modality). As well, we aim to compare this one with an orthodontic light cure composite resin. Anyway, the rationale of this research is not only to compare the two polymeric materials demonstrating which of the two is more reliable, since that they are both widely accepted and of general use. Conversely, we aimed to test in the orthodontic field the behavior of the two polymers under a particular condition, consisting of the enamel pre-etching before applying a universal adhesive. As previously reported, this latter procedure has highly improved the bonding force in restorative dentistry studies, but to our knowledge no research has been conducted in orthodontics until now.

The first two null hypotheses have been investigated in vitro to respectively show that there are no significant differences in shear bond strength (SBS) between the two resins tested, as well as in adhesive remnant index (ARI) scores. The third null hypothesis investigated through a randomized clinical trial is that no significant differences in failure and survival rates of fixed retainers bonded with the abovementioned resins occur during a 24-month follow up.

## 2. Materials and Methods

### 2.1. In Vitro Study

#### 2.1.1. Specimen Preparation

The Unit Internal Review Board approved this study. Forty freshly extracted bovine incisors were selected after meeting the inclusion criteria: integrity of the buccal surface, absence of enamel wear or caries, absence of anomalies of volume, shape and structure, and absence of traumatic lesions [24]. After being extracted, teeth were kept inside a solution of 0.1% (*w*/*v*) thymol free of alcohol, in complete darkness [25]. Within a few days, debris was removed through a scalpel and teeth were carefully cleaned using a toothbrush, rinsed, and then dried. Finally, the root of each incisor was embedded into cold-curing fast-setting acrylic (Leocryl, Leone s.p.a., Sesto Fiorentino, Italy) inside a plastic cylindrical mold (2 cm height × 2 cm diameter). Teeth were positioned at the centre of the respective mold, equidistant from its borders, with an axis of insertion allowing a shearing force to act on the vestibular enamel.

For the adhesion, the universal adhesive Scotchbond Universal (3 M, St. Paul, MN, USA) was used according to the total-etch modality. The buccal enamel was etched for 30 s with 37% orthophosphoric acid (Gerhò Etchant gel 37%, Gerhò spa, Terlano, Italy) which was then rinsed thoroughly for other 30 s. On a surface of 3 mm^2^, corresponding to the site of application of the fixed retainer, the adhesive was immediately applied with a brush, rubbed for 5 s, gently air-dried for 4 s allowing the solvent to evaporate, and finally light cured for 20 s with an LED unit (Starlight Pro, Mectron s.p.a., Carasco, Italy). 

A 1-mm thick and round section wire (Ortosmail Krugg, Milan, Italy) was cut in parts of 3-millimeter length and a single portion was applied on each sample. From now on, teeth were randomly divided into two groups. In group 1 (control, *n* = 20 samples), the orthodontic light curing composite resin Transbond XT (3M, St. Paul, MN, USA) was applied around the retainers. Conversely, as to group 2 (trial, *n* = 20 samples) (FL), this procedure was performed with Filtek Supreme XTE (3 M, St. Paul, MN, USA), a flowable nanocomposite commonly used for restorations, sealings, and repair of resin or acrylic temporaries. This experimental group is not conventional.

A probe was used to remove the excess of the materials in order not to exceed the area of 3 mm^2^. Finally, resins were light cured for 10 s in an occlusal-apical direction and 10 s in the opposite one. 

Transbond XT is a dental resin, which is based on bis-GMA as polymeric matrix. It is highly filled since it contains 70% to 80% of silane treated quartz. Conversely, Filtek Supreme XTE is a flowable nanocomposite, again with a bis-GMA-based polymeric network but with a considerably lower percentage of filler. 

Table 1 lists the properties of the materials tested in this study along with the protocols recommended for their application. 

#### 2.1.2. Shear Bond Strength (SBS) Test

**Figure 3:**
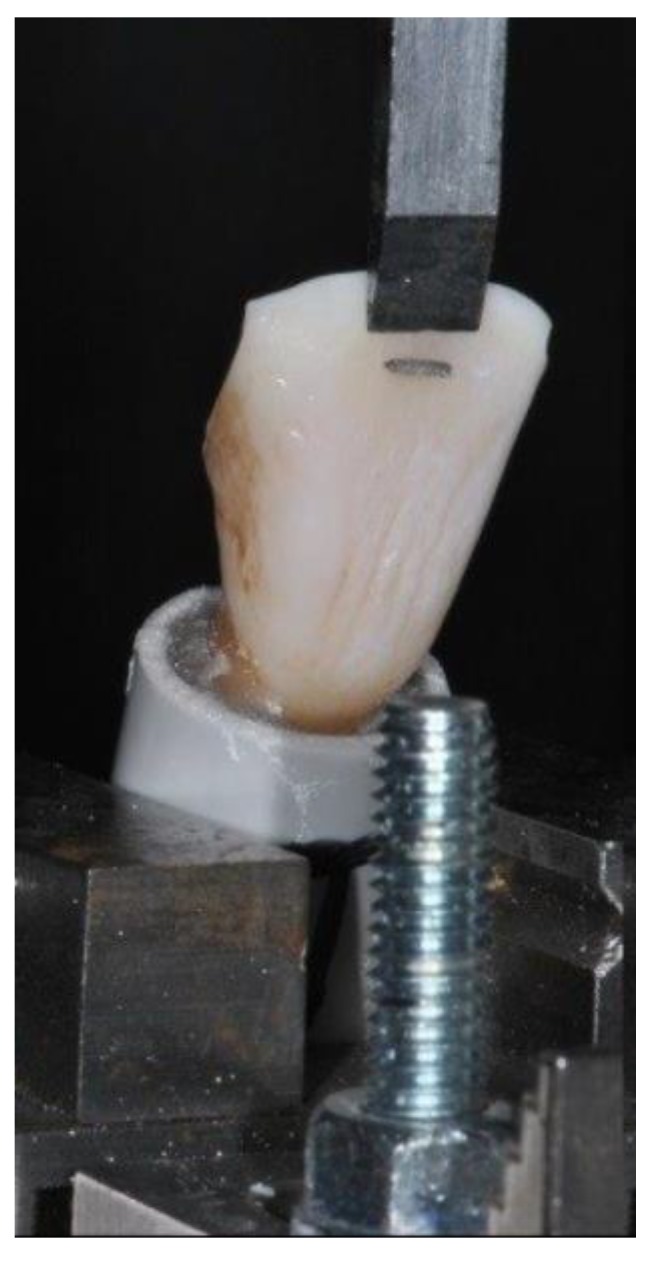
Positioning of specimens into the universal testing machine (three-quarter view).

The preparation of the samples described above and the subsequent SBS test were performed according to previous studies [11,24]. Each sample was secured in the lower jaw of a universal testing machine (Model 3343, Instron, Canton, MA, USA) keeping the long axis of the splints parallel to the edge of the blade. These were stressed with a steel tip acting in an occluso-gingival direction, tangentially to the adhesion surface, until their detachment, as shown in Figure 3. The crosshead speed was set at 1 mm/min [26]. The maximum load required to debond the wires was automatically recorded in newtons (N), using the software Bluehill 2 (Instron Industrial Products, Grove City, Pennsylvania, PA, USA). Data were converted into megapascals (MPa) as the ratio of newtons to surface area.

#### 2.1.3. Adhesive Remnant Index (ARI) Score

Enamel was examined at ×10 magnification with the use of a microscope (Stereomicroscope SR, Zeiss, Oberkochen, Germany) in order to determine the amount of adhesive remaining on the enamel of each tooth after the detachment of the splints [27]. This parameter was determined according to the ARI scale ranging from 0 to 3 (0: no adhesive; 1: less than 50%; 2: more than 50%; and 3: 100% adhesive) in order to define the bond failure site [28,29].

### 2.2. Randomized Clinical Trial (RCT) 

#### 2.2.1. Trial Design 

Even this study has obtained the approval of the Unit Internal Review Board. It consisted of a parallel-group, randomized, active controlled, and single-center trial with a 1:1 allocation ratio. As to the methods, no variations occurred during the trial.

#### 2.2.2. Participants

Patients addressing to the Unit of Orthodontics and Paediatric Dentistry, Section of Dentistry, Department of Clinical, Surgical, Diagnostic and Paediatric Sciences, University of Pavia, Pavia, Italy, were recruited from June 2016 to January 2018 and the study lasted until January 2020. The consent of participants, or that of parents in case of underage patients, was required. Both interventions and outcome assessment were conducted in the abovementioned center.

**Figure 4:**
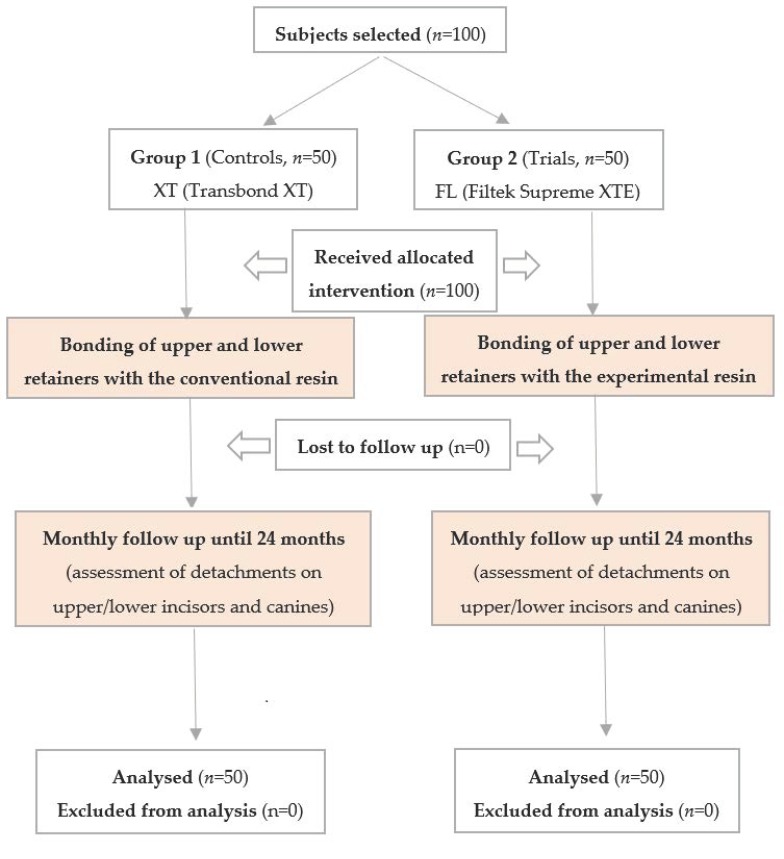
Flow chart showing participants and the protocol used in this study.

The inclusion criterion was being at the end of an orthodontic treatment requiring the subsequent application of a lingual and palatal fixed retainer, whereas reported facial trauma, onychophagia and habit of biting pencils or pens constituted exclusion criteria. The flow chart of this study is shown in Figure 4.

#### 2.2.3. Interventions

When each participant completed the orthodontic treatment, an upper and lower canine-to-canine retainer was applied. Participants were randomly divided into two groups, according to the composite resin subsequently used for the retainer adhesion. After enamel etching and application of Scotchbond Universal adhesive, in the first arm the orthodontic composite resin Transbond XT was positioned around the retainer, whereas in the second arm this action was performed using the flowable nanocomposite Filtek Supreme XTE. The abovementioned materials are the same previously tested in the in vitro study. No other variables were considered between the two groups. In order to standardize the procedure, interventions have been always carried out by the same operator.

#### 2.2.4. Outcomes

Since the moment of the two retainers bonding, each participant underwent a 24-month follow up with monthly visits, realized by an operator not previously involved in the splint bonding, nor aware of the in vitro tests. Each detachment was registered, distinguishing both the arch (maxillary and mandibular) and the teeth involved (incisors and canines). After the first failure, rebounded teeth were not further included in the statistical analysis. Participants were asked to strictly respect the scheduled appointments and to immediately inform the orthodontist if suspecting any detachment. No variations to the outcome occurred after the trial commencement.

#### 2.2.5. Sample Size 

Sample size calculation (Alpha = 0.05; Power = 90%) for two independent study groups and a continuous primary endpoint required 100 total participants (45.5% males, mean age 25 years and 2 months; 54.5% females, mean age 23 years and 9 months): 50 controls (47% males, mean age 26 years and 2 months; 53% females, mean age 25 years and 1 month) and 50 trials (44% males, mean age 24 years and 2 months; 56% females, mean age 22 years and 5 months), corresponding to a total of 1200 teeth splinted. A total of 106 patients were visited before the beginning of the study, but 4 refused to participate and 2 did not meet the inclusion criteria. One hundred final subjects when then selected, as requested by the sample size calculation. Interim analysis and stopping guidelines were not applicable. 

#### 2.2.6. Randomization and Blinding 

Using a block randomization table, the data analyst provided a randomization sequence, considering a permuted block of fifty participants. The operator who enrolled participants also allocated them to the intervention using sequentially numbered and sealed envelopes with the allocation cards previously prepared. Blinding him was not technically possible. Conversely, participants, data assessor, and data analyst were always blinded during the study because none of them knew the treatment administered to each participant. No visible differences between retainers bonded with the two different methods could be noticed when assessing the outcomes. 

### 2.3. Statistical Methods 

Data were submitted to statistical analysis with R Software (R version 3.1.3, R Development Core Team, R Foundation for Statistical Computing, Wien, Austria) [30]. Significance for all statistical tests was predetermined at *P* < 0.05. 

For SBS values, descriptive statistics (mean, standard deviation, minimum, median, and maximum values for each group) were calculated. Data normality was calculated using the Kolmogorov–Smirnov test. The t-test was applied to determine the existence of significant differences in detachment forces between the two groups. As regards ARI scores, a frequency analysis by means of the χ^2^ test was conducted to assess significant differences between the groups. 

Data assessed within the RCT underwent a Fisher exact test to detect differences among the frequencies of clinical detachments of the groups tested; finally, the respective Kaplan-Meier survival curves of the two resins tested were constructed and compared using the log-rank test.

## 3. Results

### 3.1. In Vitro Study

#### 3.1.1. Shear Bond Strength (SBS) Test

As shown in Table 2, group 2 (FL) showed a significant lower mean detachment force, if compared to group 1 (XT) (t-test, *P* < 0.05).

#### 3.1.2. Adhesive Remnant Index (ARI) Score

No significant differences between the groups have been shown by the χ^2^ test (*P* > 0.05). Both group 1 and group 2 reported a major frequency of ARI scores “1” and “2”, as shown in Table 3.

### 3.2. Randomized Clinical Trial (RCT)

After the 24-month follow up, statistically significant differences in the detachment rate were found between group 1 and group 2 (*P* < 0.05). In fact, for both the upper and lower arch, as well as if considering them overall, significantly higher total failures were found in the trial group (Group 2), as shown in Table 4. Moreover, within this group, the lower teeth splinted reported a higher failure rate (*P* < 0.05) if compared to the upper ones (13.33% vs. 10.67%) and this difference was evident also within group 1 (7.00% and 5.33%) but in both cases without statistical significance (*P* > 0.05). 

If comparing the bonding performance on incisors and canines, as shown in Table 5, no statistically significant difference was detected in failure rates, independently of the group and of the arch considered (*P* > 0.05), despite higher detachments for incisors.

**Figure 5:**
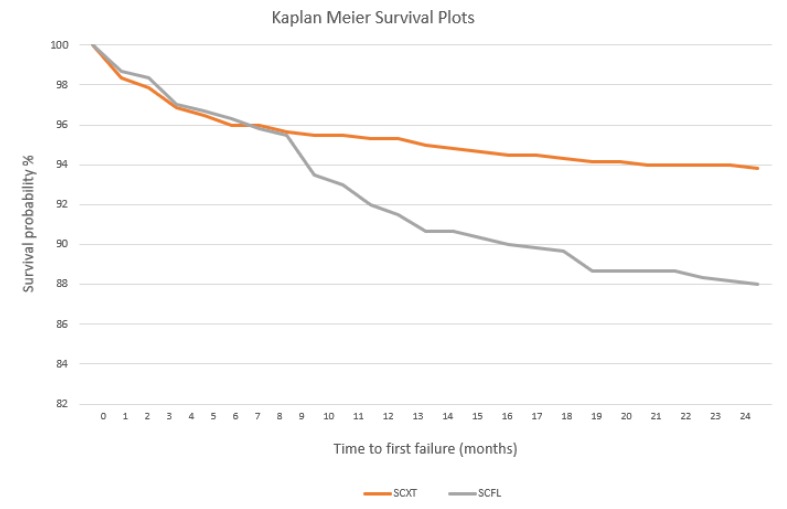
Kaplan-Meier survival curves of retainers bonded with the two different resins.

Kaplan-Meier survival curves of retainers bonded with the two protocols are showed in Figure 5. Considering the interval of 0–6 months from the application of the splints, no significant difference was assessed between the groups, which reported an analogue survival probability decrease. Conversely, a statistically major failure risk was assessed for group 2 after 6 months (Hazard Ratio: 0.50; 95% Confidence Interval: 0.34%–0.72%; log rank test: 0.0002), until the end of the follow up (24 months). 

## 4. Discussion

The results achieved through an orthodontic treatment might unfortunately not be stable considering the potentiality of teeth to recover the previous position [31]. Therefore, retention is essential whether the patient desires to maintain the effects achieved, and with this purpose, fixed retainers have gained importance in relapse prevention [32,33]. The most recently proposed in dental practice are made of fiber-reinforced resin composite (FRC) [34,35] which guarantees high mechanical properties and good aesthetic effects [36]. However, further studies should be conducted in the future analyzing FRC wires’ mechanical behavior [37]. 

Nowadays, multistranded flexible spiral wire retainers are considered the gold standard in orthodontics [38]. Manufacturers have introduced plenty of resins that can be used for bonding procedures. The rationale of this study was to assess both the efficacy and the reliability over time of the flowable nanocomposite Filtek Supreme XTE, generally used in restorative dentistry, and to compare this with the common orthodontic resin Transbond XT.

The first null hypothesis of this study was rejected. In fact, if compared to the control resin, the experimental one showed in vitro a significantly lower SBS. This can be explained considering the different composition of the two materials tested since the latter belongs to flowable composite resins, which are made of a lower percentage of filler. In fact, the amount of this component is strictly related to the hardness of the material, as well as to the resistance to abrasion and fracture; therefore, flowable resins generally show lower mechanical properties [39]. According to our results, both Aldrees et al., [40] and Reicheneder et al., [41] found a higher bonding force when comparing Transbond LR to flowable resins, independently of the wire associated.

However, Radlanski and Zain [42] showed that, despite no significant differences, Tetric Flow (microfilled hybrid) reported higher bond strength values than Heliosit Orthodontic (microfilled). Anyway, in accordance with our results, this is due to the higher filler content and the superior tensile and bending strengths of Tetric Flow. In the study of Tabrizi et al., [43] the major SBS values were strangely assessed for Filtek Supreme, but significant differences neither occurred with the orthodontic highly filled composite Light Bond, nor with the flowable composites Tetric Flow and FlowTain. Moreover, the mean SBS value obtained for Filtek Supreme in the abovementioned study is notably higher than the one assessed in our report for the same material (22.4 MPa vs. 8.28 MPa), but this can more likely be explained considering the different light curing times that followed (40 s vs. 20 s). 

The second null hypothesis of this study was rejected. The assessment of ARI scores is important in order to define the bonding failure site. The two groups reported no significant differences between them and a major frequency of ARI “1” and “2” was noticed, corresponding respectively to less or more than 50% of adhesive remnant on enamel. Most authors generally report a cohesive fracture with a failure at the wire–composite interface [21,22], although in other studies the enamel–composite interface was the most frequently affected [42]. In this report, no conclusive considerations can be advanced due to the assessment of both ARI “1” and “2” without significant differences. Anyway, no “0” ARI scores were assessed in this study, which confirms the efficacy of a universal adhesive used in total etch modality.

The values obtained are ideal since they indicate a good adhesion of the tested materials to teeth, and at the same time, a facilitated enamel finishing after debonding. Moreover, bonding strength values are included in the interval of 5–50 MPa, which is considered a valid range for materials to support masticatory forces [44]. In sight of these results obtained in vitro, both adhesives appear reliable, although a significantly major shear force is assessed for the conventional resin. 

A limitation of this in vitro study is that bovine teeth were used, due to the difficulty in obtaining human ones for research purposes. Although they differ considering their geometry and size, similar compositions and physical properties have been described between the two, allowing bovine teeth to be used as valid substitutes in bonding tests [27,45]. Furthermore, results of in vitro studies might not be directly transferred to a clinical setting because of the different conditions to which materials are exposed in vivo. For this reason, we aimed to assess whether a different failure and survival rate occurs over time considering splints bonded with XT and FL. 

Considering the clinical study, the third null hypothesis was rejected. In fact, in accordance with the in vitro SBS test, significantly higher failures were assessed after a 24-month follow up for splints bonded with the experimental resin, considering both the maxillary and mandibular arch, as well as them overall. Within each group considered, the lower teeth splinted reported a higher failure rate with respect to the upper ones, but this difference was not statistically relevant in any group. Although this is in accordance with other studies [46], controversial results have been reported about the detachments of orthodontic fixed retainers in upper and lower arches [47]. 

Since in both groups the same universal adhesive in total-etch modality has been used, no influence can be attributed to this component as regards the differences assessed. However, in a previous clinical study where lower retainers have been bonded with Transbond XT resin [48], the number of detachments assessed was greater. This can be explained considering that Transbond XT primer was used, instead of Scotchbond Universal.

Another aim of this research was to assess whether incisors and canines show a different pattern in bonding failure. In fact, some authors report that canine-and-canine retainers are more reliable than those bonded to all anterior teeth, despite a major discomfort and risk of relapse of unbonded incisors [33]. Failures on incisors might be more frequent than those happening on canines. Although this event has been verified in this report, no statistically significant differences between the elements were detected, independently of the group and of the arch considered (*P* > 0.05).

Finally, the third purpose was to compare the survival rate of the two groups during a 24-month follow up, by means of Kaplan-Meier survival curves. No differences were shown until the first 6 months for retainers bonded with the two materials, and therefore the two curves are almost overlapping. However, during the following months of follow up, a clear separation between the curves occurs and at the end of 24 months. The survival rate for the groups bonded with the conventional XT and with the experimental FL are respectively 93.83% and 88%, with a significant difference between the two. According to these results, it can be stated that until the first months of retainers bonding, the different resin used does not influence the decrease of survival rate over time, probably because of the efficacy of the bonding primer used. Subsequently, however, the two resins show a different pattern of failure. The cyclic loading due to masticatory forces in the oral cavity, as well as the degradation exerted by saliva, affect the mechanical properties of resins [49]. Obviously, this phenomenon is more evident for flowable with respect to highly filled composite materials which justifies the remarkable decrease in survival rate for participants bonded with FL.

The main limitation of this clinical trial is that the results here obtained are not directly comparable since no studies of other authors have been found to date where fixed retainers were bonded with a universal adhesive in total-etch modality instead of using a conventional orthodontic primer. 

## 5. Conclusions

Considering the limitations described and according to the result obtained, we can conclude that:

In vitro, a significantly lower SBS has been obtained for retainers splinted with FL instead of the conventional orthodontic XT, due to the lower filler percentage of the former material. Both groups reported a major frequency of ARI “1” and “2”, but without statistically significant difference between the two groups.

During a 24-month clinical follow up of fixed retainers bonded in vivo respectively with the two composite resins, a lower failure rate was assessed for both arches using XT. Despite a comparable pattern in the first six months, a significantly lower survival rate is subsequently reported by retainers bonded with the FL. 

## Figures and Tables

**Table 1:** Materials used and protocol recommended for their application.

Material	Type	Composition	pH	Application Protocol
Scotchbond Universal	Universal adhesive	10-MDP, HEMA, silane, dimethacrylate resins, Vitrebond^TM^ copolymer, filler, ethanol, water, initiators, catalysts	2.7	According to the total-etch modality: 1. Tooth isolation 2. Etching 3. Rinsing and air-drying 4. Adhesive application 5. Air-drying 6. Light curing for 20”
Transbond XT	Orthodontic light curing composite resin	Silane treated quartz (70%-80%), Bis-GMA (10%-20%), Bisphenol A Bis(2-hydroxyethyl ether) dimethacrylate (5%-10%), Silane treated silica (<2%), DPIHFP (<0.2%)	-	1. Apply around lingual retainer 2. Light curing for 20”
Filtek Supreme XTE	Flowable light curing nanocomposite	bis-GMA, UDMA, TEGDMA, bis-EMA(6)	-	1. Apply around lingual retainer 2. Light curing for 20”

Legend: 10-MDP, 10-Methacryloyloxydecyl dihydrogen phosphate; HEMA, 2-hydroxyethyl methacrylate; bisGMA, Bisphenol A diglycidyl ether dimethacrylate; DPIHFP, Diphenyliodonium hexafluorophosphate; UDMA, urethane dimethacrylate; TEGDMA: Triethylene glycol dimethacrylate; bis-EMA, Ethoxylated bisphenol A dimethacrylate.

**Table 2:** Detachment forces (MPa) of the two resins tested.

Group	Resin	Mean	SD	Minimum	Median	Maximum	Significance
							
1 (XT)	Conventional	13.31	2.87	8.95	12.85	21.64	
2 (FL)	Flowable	8.28	1.45	5.54	8.22	11.80	*P* < 0.05

SD: standard deviation.

**Table 3:** Frequencies of adhesive remnant index (ARI) indexes for both groups.

Score	Group 1 (XT)	Group 2 (FL)
ARI = 0	0	0
ARI = 1	35	45
ARI = 2	60	45
ARI = 3	5	10

**Table 4:** Numbers and failure rates of fixed retainers for the overall, upper, and lower teeth in the two groups tested.

Group	Composite	Splint Zone	Teeth Bonded	Failures	Percentage (%)	Significance
Group 1	Conventional	Overall	600	37	6.17	
Group 2	Flowable	Overall	600	72	12.00	*P* < 0.05
Group 1	Conventional	Upper	300	16	5.33	
Group 2	Flowable	Upper	300	32	10.67	*P* < 0.05
Group 1	Conventional	Lower	300	21	7.00	
Group 2	Flowable	Lower	300	40	13.33	*P* < 0.05

**Table 5:** Numbers and failure rates of fixed retainers for upper and lower teeth in the two groups tested, distinguishing canines and incisors.

Group	Composite	Splint Zone	Tooth	Teeth Bonded	Failures	Percentage (%)	Significance
Group 1	Conventional	Upper	Canines	100	2	2.00	
Group 1	Conventional	Upper	Incisors	200	14	7.00	ns
Group 1	Conventional	Lower	Canines	100	4	4.00	
Group 1	Conventional	Lower	Incisors	200	17	8.50	ns
Group 2	Flowable	Upper	Canines	100	7	7.00	
Group 2	Flowable	Upper	Incisors	200	25	12.50	ns
Group 2	Flowable	Lower	Canines	100	9	9.00	
Group 2	Flowable	Lower	Incisors	200	31	15.50	ns

## References

[1] Littlewood S.J., Millett D.T., Doubleday B., Bearn D.R., Worthington H.V. (2016). Retention procedures for stabilising tooth position after treatment with orthodontic braces. Cochrane Database Syst. Rev..

[2] Gugger J., Pandis N., Zinelis S., Patcas R., Eliades G., Eliades T. (2016). Retrieval analysis of lingual fixed retainer adhesives. Am. J. Orthod. Dentofac. Orthop..

[3] Lai C.S., Grossen J.M., Renkema A.M., Bronkhorst E., Fudalej P.S., Katsaros C. (2014). Orthodontic retention procedures in Switzerland. Swiss Dent. J..

[4] Renkema A.M., Sips E.T., Bronkhorst E., Kuijpers-Jagtman A.M. (2009). A survey on orthodontic retention procedures in the Netherlands. Eur. J. Orthod..

[5] Singh P., Grammati S., Kirschen R. (2009). Orthodontic retention patterns in the United Kingdom. J. Orthod..

[6] Vandevska-Radunovic V., Espeland L., Stenvik A. (2013). Retention: Type, duration and need for common guidelines. A survey of Norwegian orthodontists. Orthodontics.

[7] Valiathan M., Hughes E. (2010). Results of a survey-based study to identify common retention practices in the United States. Am. J. Orthod. Dentofac. Orthop..

[8] Wong P.M., Freer T.J. (2004). A comprehensive survey of retention procedures in Australia and New Zealand. Aust. Orthod. J..

[9] Scribante A., Gandini P., Tessera P., Vallittu P.K., Lassila L., Sfondrini M.F. (2017). Spot-Bonding and Full-Bonding Techniques for Fiber Reinforced Composite (FRC) and Metallic Retainers. Int. J. Mol. Sci..

[10] Batra C., Nagpal R., Tyagi S.P., Singh U.P., Manuja N. (2014). In Vitro bonding effectiveness of three different one-step self-etch adhesives with additional enamel etching. J. Investig. Clin. Dent..

[11] Beltrami R., Chiesa M., Scribante A., Allegretti J., Poggio C. (2016). Comparison of shear bond strength of universal adhesives on etched and nonetched enamel. J. Appl. Biomater. Funct. Mater..

[12] De Goes M.F., Shinohara M.S., Freitasc M.S. (2014). Performance of a New One-step Multi-mode Adhesive on Etched vs Non-etched Enamel on Bond Strength and Interfacial Morphology. J. Adhes. Dent..

[13] Erickson R.L., Barkmeier W.W., Latta M.A. (2009). The role of etching in bonding to enamel: A comparison of self-etching and etch-and-rinse adhesive systems. Dent. Mater..

[14] Khosravi K., Ataei E., Mousavi M., Khodaeian N. (2009). Enamel Margins on the Microleakage of a Simplified All-in-One and a Self-etch Adhesive System. Oper. Dent..

[15] Lührs A.K., Guhr S., Schilke R., Borchers L., Geurtsen W., Günay H. (2008). Shear Bond Strength of Self-etch Adhesives to Enamel with Additional Phosphoric Acid Etching. Oper. Dent..

[16] Manuja N., Nagpal R., Pandit I.K. (2012). Dental Adhesion: Mechanism, Techniques and Durability. J. Clin. Pediatr. Dent..

[17] Miguez P.A., Castrob P.S., Nunesb M.F., Waltera R., Pereirac P.N.R. (2003). Effect of Acid etching on the Enamel Bond of Two Self-etching Systems. Adhes. Dent..

[18] Suzuki T., Takamizawa T., Barkmeier W.W., Tsujimoto A., Endo H., Erickson R.L., Latta M.A., Miyazaki M. (2016). Influence of Etching Mode on Enamel Bond Durability of Universal Adhesive Systems. Oper. Dent..

[19] Van Landuyt K.L., Kanumilli P., De Munck J., Peumans M., Lambrechts P., Van Meerbeek B. (2006). Bond strength of a mild self-etch adhesive with and without prior acid-etching. J. Dent..

[20] Van Meerbeek B., Kanumilli P., De Munck J., Van Landuyt K., Lambrechts P., Peumans M. (2005). A randomized controlled study evaluating the effectiveness of a two-step self-etch adhesive with and without selective phosphoric-acid etching of enamel. Dent. Mater..

[21] Cooke M.E., Sherriff M. (2010). Debonding force and deformation of two multi-stranded lingual retainer wires bonded to incisor enamel: An in Vitro study. Eur. J. Orthod..

[22] Milheiro A., de Jager N., Feilzer A.J., Kleverlaan C.J. (2015). In Vitro debonding of orthodontic retainers analyzed with finite element analysis. Eur. J. Orthod..

[23] Oesterle L.J., Shellhart C., Henderson S. (2001). Enhancing wire-composite bond strength of bonded retainers with wire surface treatment. Am. J. Orthod. Dentofac. Orthop..

[24] Scribante A., Dermenaki Farahani M.R., Marino G., Matera C., Rodriguez Y Baena R., Lanteri V., Butera A. (2020). Biomimetic Effect of Nano-Hydroxyapatite in Demineralized Enamel before Orthodontic Bonding of Brackets and Attachments: Visual, Adhesion Strength, and Hardness in In Vitro Tests. Biomed. Res. Int..

[25] Scribante A., Sfondrini M.F., Fraticelli D., Daina P., Tamagnone A., Gandini P. (2013). The influence of no-primer adhesives and anchor pylons bracket bases on shear bond strength of orthodontic brackets. Biomed. Res. Int..

[26] Colombo M., Gallo S., Padovan S., Chiesa M., Poggio C., Scribante A. (2020). Influence of Different Surface Pretreatments on Shear Bond Strength of an Adhesive Resin Cement to Various Zirconia Ceramics. Materials (Basel).

[27] Poggio C., Scribante A., Della Zoppa F., Colombo M., Beltrami R., Chiesa M. (2014). Shear bond strength of one-step self-etch adhesives to enamel: Effect of acid pretreatment. Dent. Traumatol..

[28] Artun J., Bergland S. (1984). Clinical trials with crystal growth conditioning as an alternative to acid-etch enamel pretreatment. Am. J. Orthod..

[29] Scribante A., Cacciafesta V., Sfondrini M.F. (2006). Effect of various adhesive systems on the shear bond strength of fiber-reinforced composite. Am. J. Orthod. Dentofac. Orthop..

[30] R Development Core Team (2008). R: A Language and Environment for Statistical Computing.

[31] Sfondrini M.F., Fraticelli D., Castellazzi L., Scribante A., Gandini P. (2014). Clinical evaluation of bond failures and survival between mandibular canine-to-canine retainers made of flexible spiral wire and fiber-reinforced composite. J. Clin. Exp. Dent..

[32] Dahl E.H., Zachrisson B.U. (1991). Long-term experience with direct-bonded lingual retainers. J. Clin. Orthod..

[33] Stormann I., Ehmer U. (2002). A prospective randomized study of different retainer types. J. Orofac. Orthop..

[34] Scribante A., Vallittu P.K., Özcan M. (2018). Fiber-Reinforced Composites for Dental Applications. Biomed. Res. Int.

[35] Freilich M.A., Karmaker A.C., Burstone C.J., Goldberg A.J. (1998). Development and clinical applications of a light-polymerized fiber-reinforced composite. J. Prosthet. Dent..

[36] Vallittu P.K., Lassila V.P. (1992). Reinforcement of acrylic resin denture base material with metal or fibre strengtheners. J. Oral Rehabil..

[37] Sfondrini M.F., Vallittu P.K., Lassila L.V.J., Viola A., Gandini P., Scribante A. (2020). Glass Fiber Reinforced Composite Orthodontic Retainer: In Vitro Effect of Tooth Brushing on the Surface Wear and Mechanical Properties. Materials (Basel).

[38] Tacken M.P.E., Cosyn J., De Wilde P., Aerts J., Govaerts E., Vannet B.V. (2010). Glass fiber reinforced versus multistranded bonded orthodontic retainers: A 2 year prospective multi-centre study. Eur. J. Orthod..

[39] Karaman A.I., Polat Ö., Büyükyilmaz T. (2003). A practical method of fabricating a lingual retainer. Am. J. Orthod. Dentofac. Orthop..

[40] Aldrees A.M., Al-Mutairi T.K., Hakami Z.W., Al-Malki M.M. (2010). Bonded orthodontic retainers: A comparison of initial bond strength of different wire-and-composite combinations. J. Orofac. Orthop..

[41] Reicheneder C., Hofrichter B., Faltermeier A., Proff P., Lippold C., Kirschneck C. (2014). Shear bond strength of different retainer wires and bonding adhesives in consideration of the pretreatment process. Head Face Med..

[42] Radlanski R.J., Zain N.D. (2004). Stability of the Bonded Lingual Wire Retainer—A Study of the Initial Bond Strength. J. Orofac. Orthop..

[43] Tabrizi S., Salemis E., Usumez S. (2010). Flowable Composites for Bonding Orthodontic Retainers. Angle Orthod..

[44] Scribante A., Contreras-Bulnes R., Montasser M.A., Vallittu P.K. (2016). Orthodontics: Bracket Materials, Adhesives Systems, and Their Bond Strength. Biomed. Res. Int..

[45] Soares F.M.Z., Follak A., da Rosa L.S., Montagner A.S., Lenzi T.L., Rocha R.O. (2016). Bovine tooth is a substitute for human tooth on bond strength studies: A systematic review and meta-analysis of in vitro studies. Dent. Mater..

[46] Salehi P., Zarif Najafi H., Roeinpeikar S.M. (2013). Comparison of survival time between two types of orthodontic fixed retainer: A prospective randomized clinical trial. Prog. Orthod..

[47] Kocher K.E., Gebistorf M.C., Pandis N., Fudalej P.S., Katsaros C. (2019). Survival of maxillary and mandibular bonded retainers 10 to 15 years after orthodontic treatment: A retrospective observational study. Prog. Orthod..

[48] Scribante A., Sfondrini M.F., Broggini S., D’Allocco M., Gandini P. (2011). Efficacy of Esthetic Retainers: Clinical Comparison between Multistranded Wires and Direct-Bond Glass Fiber-Reinforced Composite Splints. Int. J. Dent..

[49] Drummond J.L. (2008). Degradation, fatigue, and failure of resin dental composite materials. J. Dent. Res..

